# Yttrium-90 radioembolization for colorectal cancer liver metastases: a prospective cohort study on circulating angiogenic factors and treatment response

**DOI:** 10.1186/s13550-016-0236-1

**Published:** 2016-12-21

**Authors:** C. E. N. M. Rosenbaum, A. F. van den Hoven, M. N. G. J. A. Braat, M. Koopman, M. G. E. H. Lam, B. A. Zonnenberg, H. M. Verkooijen, M. A. A. J. van den Bosch

**Affiliations:** 1 Department of Radiology and Nuclear Medicine, University Medical Center Utrecht, Room E.01.132, Heidelberglaan 100, 3584 CX Utrecht, The Netherlands; 2 Department of Medical Oncology, University Medical Center Utrecht, Utrecht, The Netherlands

**Keywords:** Yttrium-90 radioembolization, Colorectal cancer liver metastases, Angiogenesis

## Abstract

**Background:**

Yttrium-90 radioembolization (^90^Y-RE) as a treatment for liver tumours induces radiation damage and hypoxia in liver tissue, which is also a trigger for systemic release of angiogenic factors, potentially stimulating tumour growth. We examined changes in circulating angiogenic factors following ^90^Y-RE and investigated the association between response and angiogenic factors. In this prospective study, 42 patients with unresectable, chemorefractory metastatic colorectal cancer (CRCLM) were treated with ^90^Y-RE. Blood samples were collected pre-treatment and at 0, 1, 3, 7 and 30 days of follow-up. Response was measured with MRI according to RECIST 1.1 at 1 month and subsequently 3-month interval until progressive disease (PD) occurred. Associations between circulating angiogenic factors and response were examined with linear mixed model analysis.

**Results:**

Following ^90^Y-RE, three angiogenic factors demonstrated an increase in plasma levels, i.e., vascular endothelial growth factor (VEGF), hepatocyte growth factor (HGF) and angiopoietin-2 (Ang-2). Non-responders (= PD at 1-month follow-up, *n* = 10) had a significant increase of Ang-2 and HGF at 3 and 7 days post treatment compared to responders (= stable disease or better, *n* = 32), who showed little to no changes in plasma levels (respectively *p* = 0.01 and *p* = 0.007). Median overall survival was 9.2 months (95% confidence interval 6.1–12.4).

**Conclusions:**

Significant increases in plasma levels of Ang-2 and HGF in the first week after treatment were associated with rapid progressive disease of liver lesions at 1 month after ^90^Y-RE. Combination of ^90^Y-RE with anti-angiogenic therapy may reduce these effects and result in better response.

## Background

Yttrium-90 radioembolization (^90^Y-RE) is an intra-arterial treatment option for patients with liver dominant, unresectable and chemorefractory hepatic malignancies. Microspheres with a diameter of 30–40 μm are embedded with the radio-isotope yttrium-90 (^90^Y) and delivered to the liver via a catheter in the hepatic artery. These microspheres will travel distally with the blood stream and lodge at the arteriolar level inside the tumours and normal liver and cause tumour necrosis through radiation and embolic effects [1–5].

A possible consequence of locoregional treatment with ^90^Y-RE is the systemic release of angiogenic growth factors due to the embolic effect of this treatment, which can induce growth of untreated lesions or extrahepatic (micro)metastases and potentially affect patient survival. A systemic release of growth factors after resection of the primary colon tumour has already been suggested based on an increased metabolic activity in liver metastases [6, 7]. Furthermore, in patients with hepatocellular carcinoma (HCC), a rise in circulating levels of vascular endothelial growth factor (VEGF) has been described following trans arterial chemoembolization (TACE) as well as after trans arterial bland embolization [8, 9]. Also, serum levels of VEGF were significantly different between responders and non-responders [8, 10].

Although ^90^Y-RE is a promising palliative treatment option for patients with colorectal cancer liver metastases (CRCLM), not all patients experience good response and some patients show rapid progression of liver lesions or of extrahepatic lesions. However, few large studies have been conducted in this specific patient group and by extension little prospectively collected evidence on predictive factors for outcome is available [11]. The variable response rates may be related to an increase in circulating growth factors, or even to a higher baseline level of these factors prior to treatment [12–14]. If an upregulation of angiogenic factors is related to response, then concomitant use of anti-angiogenic treatment with radioembolization may improve patient outcome.

We conducted a prospective cohort study in patients with colorectal cancer liver metastases treated with ^90^Y-RE (resin microspheres) and measured several circulating angiogenic factors at baseline and at several intervals after treatment. Aims of the study were (1) to examine changes in serum levels of several angiogenic factors following ^90^Y-RE and (2) to investigate the relationship between plasma levels of these factors and treatment response of liver lesions after ^90^Y-RE.

## Methods

### Patients

Patients with unresectable and chemorefractory colorectal cancer liver metastases were enrolled in this prospective cohort study, the RADAR study (RADioembolization: Angiogenic factors and Response). This study was approved of by the institutional review board (medical ethical committee NL34970.041.11 protocol number 11-172/E). All patients gave written informed consent to participate in the study and for the study results to be published.

### Treatment and follow-up

**Fig. 1:**
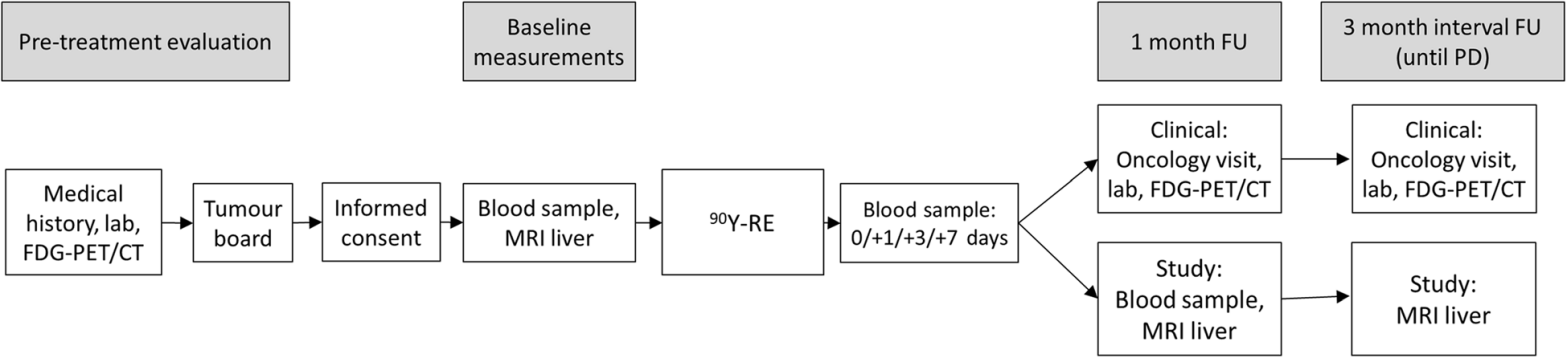
Outline of clinical evaluations and study procedures of the RADAR study. ^*90*^ *Y-RE* yttrium-90 radioembolization, *FU* follow-up, *PD* progressive disease

An overview of all pre-treatment and follow-up procedures is given in Fig. 1. All patients referred to our centre for ^90^Y-RE were screened for eligibility with full medical history, standard laboratory evaluation (including but not limited to liver function tests) and ^18^F-FDG-PET/CT imaging (including diagnostic multiphasic contrast enhanced abdominal and thoracic CT). Consensus on treatment was reached by a tumour board consisting of physicians from the department of medical oncology, department of interventional radiology and department of nuclear medicine. If there was any doubt about possible resectable liver disease (e.g. single lesion or multiple lesions but limited to one liver lobe), a liver surgeon was consulted additionally.

Yttrium-90 radioembolization was performed by experienced interventional radiologists in concordance with international guidelines [15–18]. Vascular anatomy was mapped during pre-treatment angiography. Subsequently, technetium-99 m macro aggregated albumin (^99m^Tc-MAA) particles were infused and the intrahepatic and possible extrahepatic distribution of those was evaluated with SPECT/CT imaging. In a second angiographic procedure the yttrium-90 microspheres (SIR-Spheres, SIRTeX Medical Limited, Sydney, Australia) were infused with the catheter in a similar position as during pre-treatment angiography. The injected activity was calculated using the body surface area (BSA) method in all patients. Distribution of yttrium-90 microspheres after treatment was evaluated with ^90^Y-PET imaging.

Baseline blood sample measurements were performed prior to treatment. Follow-up blood sampling for angiogenic factors was performed at days 0 (immediately after injection of the microspheres), 1, 3, 7 and 30 after ^90^Y-RE treatment. Blood samples consisted of EDTA and citrate samples. Platelet free plasma was obtained from the EDTA sample prior to rapid storage at −80 °C (within 1 h from sample collection).

Treatment response of liver lesions was evaluated with RECIST 1.1 on magnetic resonance imaging of the liver, including post-gadolinium series and diffusion-weighted imaging. MRI was scored by an independent radiologist specialized in abdominal imaging. In addition, whole body FDG-PET/CT was performed and read by an independent experienced nuclear medicine physician. Tumour response was evaluated for target lesions only, as well as for all liver lesions and for the whole body.

### Analyses of angiogenic factors

The following angiogenic factors were analysed: vascular endothelial growth factor (VEGF), hepatocyte growth factor (HGF), angiopoietin-2, basic fibroblast growth factor (FGF-b), platelet-derived growth factor (PDGF-BB), stromal cell-derived factor 1 (SDF-1a) and thrombospondin-1. Measurements were performed using an in-house developed and validated multiplex immunoassay based on Luminex technology (xMAP, Luminex Austin TX USA). Samples were incubated with antibody-conjugated MagPlex microspheres (Bio-Rad) for 1 h at room temperature with continuous shaking, followed by 1-h incubation with biotinylated antibodies, and 10-min incubation with phycoerythrin-conjugated streptavidin diluted in high-performance ELISA buffer (HPE, Sanquin, the Netherlands [19, 20]). Acquisition was performed with the Bio-Rad FlexMAP3D (Bio-Rad laboratories, Hercules USA) in combination with xPONENT software version 4.2 (Luminex). Data was analysed by 5-parametric curve fitting using Bio-Plex Manager software, version 6.1.1 (Bio-Rad). Controls were analysed to assure intra-temporal consistency of the results.

### Statistical analysis

Descriptive statistics were used to explore baseline characteristics and data on response and angiogenic factors. When boxplots suggested a possible difference between strata, independent samples *t* tests were used to evaluate this. Data were evaluated for Gaussian distribution and transformed when necessary.

Overall survival was computed as the number of days from ^90^Y-RE treatment until date of death. Seven patients were still alive at study closure and survival was censored for these patients at a common date (April 8, 2015). Progression-free survival of the liver was calculated as the number of days from ^90^Y-RE until documented disease progression on imaging. Kaplan Meier estimates for survival intervals were calculated using SPSS. Comparisons of survival distributions between strata were done using a log rank test.

To estimate the association between the plasma levels of angiogenic factors, tumour response and days after treatment (longitudinal data or repeated measures), a linear mixed model was used on the logarithmic transformed values. Time points of blood sampling were entered into the model as a factor, liver response to treatment at 1-month follow-up was entered as a fixed effect (dichotomous as response: complete response (CR), partial response (PR) or stable disease (SD); no response: progressive disease (PD)), and random intercepts were used for the individual patients (independent data). An additional analysis was performed to analyse the influence of the received liver dose on the levels of angiogenic factors by adding this as a factor in the models. Liver dose was calculated as $\frac{\mathrm{administrated}\kern0.5em \mathrm{activity}\kern0.5em \left(\mathrm{MBq}\right)}{\mathrm{treated}\kern0.5em \mathrm{liver}\kern0.5em \mathrm{volume}\kern0.5em \left(\mathrm{ml}\right)}\times 50$. Model performance was evaluated with the Akaike information criterion (AIC values). Significant contribution of separate factors was tested with ANOVA (*χ* ^2^).

## Results

### Patients

A total of 49 patients were included in our study. Seven patients were not suitable for ^90^Y-RE due to uncorrectable extrahepatic deposition of ^99m^Tc-MAA on SPECT/CT (*n* = 4), unsuitable hepatic artery configuration (*n* = 1), rapid progression of disease (*n* = 1) and inadequate haemoglobin level (n = 1). A total of 42 patients were treated with ^90^Y-RE and completed study follow-up.

Mean age was 62 years (female *n* = 13, male *n* = 29) (Table 1). Most patients (*n* = 30/42 i.e. 71 %) had liver only disease, while 12 patients had one or more tumour localisations outside the liver, mainly local lymphadenopathy (mainly in the hepatoduodenal ligament). Any measurable disease according to RECIST 1.1 outside the liver was termed extrahepatic disease (EHD). In 3 patients, the primary colorectal tumour had not been surgically resected, but treated with chemotherapy and/or radiation and was no longer metabolically active on ^18^F-FDG-PET imaging. All patients had received at least one regimen of systemic treatment. Systemic treatment was stopped upon progressive disease in 25 patients and due to toxicity in 5 patients. Twelve (out of 42) patients had declined further systemic treatment after having received at least one regimen. None of the patients were using systemic anti-tumour treatment (chemotherapy or monoclonal antibodies) while treated with ^90^Y-RE. A limited number of patients had previously undergone treatment of liver lesions, mainly surgical resection. At the time of ^90^Y-RE, tumour load in the liver was <25 % in 36 patients. ^90^Y-RE was performed in one session for right and left liver lobe in 38 patients, in two session (right and left liver lobe consecutively) in two patients, and two patients had only the right lobe treated (*n* = 1 only tumour in right lobe, *n* = 1 infusion of microspheres stopped prematurely due to severe pain).

**Table 1:** Baseline characteristics

Characteristic (total number of patients *n* = 42)	
Gender (number of patients)	
Female	13
Male	29
Age (years)	
Mean (range)	62 (34–83)
ECOG performance status (number of patients)	
0	23
1	17
2	2
Extrahepatic disease (number of patients)^a^	
None	30
Lymph node	7
Lung	3
Bone	3
Local recurrence	3
Other^c^	4
Baseline level (number of patients)	
Alkaline phosphatase	
Elevated	34
Normal	8
Leucocytes	
Elevated	0
Normal	42
Primary tumour surgically resected (number of patients)	
Yes	39
No	3
Previous liver-directed treatment (number of patients)	
Segmentectomy	5
Radiofrequency/microwave ablation	4
Hemihepatectomy	3
Other^b^	3
Liver metastases (number of patients)	
Synchronous	32
Metachronous	10
kRAS status (number of patients)	
Wild type	17
Mutation	9
Unknown	16
Previous systemic therapy lines (number of patients)	
0	0
1	15
2	16
>2	11
Received bevacizumab (number of patients)	
Yes	25
No	17
Tumour load as percentage of liver volume (number of patients)	
<25%	36
26–50%	6
>50%	0
Mean % (range)	15 (1–50)
Injected activity (MBq)	1508 (670–3675)
Liver dose (Gy)^d^	44.3 (24.1–87.5)

Baseline characteristic of 42 patients

^a^Numbers add to more than 42 because some patients had extrahepatic disease at more than one site

^b^Transarterial chemoembolization *n* = 1, radiotherapy *n* = 1, open/close procedure for intended RFA *n* = 1

^c^Brain *n* = 1, adrenal gland *n* = 2, peritoneal lesion *n* = 1

^d^Assuming homogeneous distribution of administered activity

*MBq* megabecquerel, *Gy* gray

### Tumour response

Proportions of responders were calculated for target lesions, whole liver and whole body (Table 2). Even though target lesions showed disease control in all patients at 1-month follow-up (either partial response (PR) or stable disease (SD)), 10/42 (24%) of these patients had progressive disease of the liver. This was due to the appearance of new lesions in 8 patients and due to unequivocal progression of non-target lesions in 2 patients, while all target lesions showed stable disease. A total of 5/42 patients (12%) were lost to imaging follow-up, mainly due to clinically progressive disease, hampering hospital visits for study purposes. At 3-month follow-up 17 patients showed disease control of target lesions, but considering the entire liver disease control was seen in 11 patients. Partial response of the liver at 6 months after treatment was seen in 3 patients. At the level of extrahepatic lesions, disease control rates were clearly lowest, i.e. 61, 21 and 2% at 1, 3, and 6-months of follow-up, respectively.

**Table 2:** Response after ^90^Y-RE treatment

	Target lesions			Whole liver			Including extrahepatic lesions		
	1 month^a^	3 months	6 months	1 month^a^	3 months	6 months	1 month^a^	3 months	6 months
PR	5	6	3	4	6	3	4	5	1
SD	36	11	2	27	5	1	21	4	0
PD	0	5	1	10	11	2	16	13	5
Deceased	0	4	12	0	4	12	0	4	12
Lost to follow-up	0	2	5	0	2	5	0	2	5
No follow-up due to earlier PD	-	14	19	-	14	19	-	14	19
Disease control rate (PR + SD)	41/41 (100%)	17/42 (40%)	5/42 (12%)	31/41 (76%)	11/42 (26%)	4/42 (10%)	25/41 (61%)	9/42 (21%)	1/42 (2%)

^a^One-month follow-up was not performed in 1 patient, while this patient was imaged at 3 months after treatment

### Survival

Median overall survival was 9.2 months (95% confidence interval (CI) 6.1–12.4). Median time to liver progression was 3.0 months (95% CI 2.8–3.3). A significant difference in time to liver progression was observed between patients with (*n* = 12) versus without EHD (*n* = 30) at baseline (1.4 versus 3.5 months; log rank test *p* = 0.001) (Table 3).

**Table 3:** Time to progression of liver lesions and overall survival

	Number of patients	Median overall survival (days (95 % CI))	Median PFS liver (days (95 % CI))	*p* value (OS and PFS)
EHD at baseline				
Yes	12	200 (164–236)	83 (0–179)	0.081
No	30	302 (216–388)	108 (79–137)*	0.007
Metastases				
Synchronous	32	255 (151–359)	92 (82–102)	0.266
Metachronous	10	286 (60–512)	92 (88–96)	0.811
Bevacizumab treatment				
Yes	25	322 (247–397)	108 (75–141)	0.049
No	17	215 (180–250)*	92 (87–97)	0.614
kRAS status				
Wild type	17	302 (244–360)	108 (51–165)	0.074
Mutation	9	166 (75–257)	87 (0–187)	0.05^a^
Unknown	16	282 (93–470)	92 (91–93)	
Previous liver treatment				
Yes	12	286 (142–430)	86 (59–113)	0.840
No	30	255 (148–362)	92 (91–94)	0.674
Baseline serum level				
Alkaline phosphatase				
Elevated	34	249 (159–339)	274 (134–335)	0.048
Normal	8	355 (345–365)*	92 (90–94)*	0.021
Albumin				
Decreased	11	166 (94–238)	90 (72–108)	0.003
Normal	31	351 (263–439)*	92 (91–93)	0.078
Treatment setting				
Salvage setting	25	215 (137–293)	92 (86–98)	0.030
Non-salvage setting	17	358 (347–369)*	120 (43–197)	0.070
Liver response at 1 month				
CR, PR or SD	31	341 (224–458)	–	0.003
PD	10^b^	160 (122–198)*	–	–

Non-salvage setting: patient declines (further) systemic treatment or systemic treatment must be stopped due to toxicity

*Significant difference, log rank test *p* < 0.05

*PFS* progression free survival, *EHD* extrahepatic disease, *PR* partial response, *SD* stable disease, *PD* progressive disease

^a^One patient was not evaluated at 1-month follow-up but first at 3-month follow-up

^b^ *p* values only comparing wild-type and mutation groups, not for comparison with unknown kRAS status

### Circulating angiogenic factors

A total of 214 blood samples were analysed in 42 patients over 6 time points. In two patients, the blood sample directly after injection of the microspheres (+0 days) was not collected, due to symptoms of postembolization syndrome directly following ^90^Y-RE treatment. Most missing values occurred at three and seven days after treatment (respectively 16 and 12 missing), as patients were at that moment often not able to visit the hospital due to complaints of fever and fatigue, as part of the postembolization syndrome. At 30-day follow-up, blood samples were not collected in 4 patients, 2 due to severe illness from probable radiation induced liver disease (REILD) and 2 were lost to follow-up.

A range of normal values for these factors is not available. We observed large variation in baseline angiogenic factor levels between patients. Baseline levels of angiopoietin-2 were higher in patients with median overall survival (OS) <6 months (median 566.65 pg/ml, IQR 470.23–969.65 pg/ml) compared with those with median OS >6 months (median 280.81 pg/ml, IQR 219.17–563.69 pg/ml) (*p* = 0.048). Patients with extrahepatic disease at baseline had significantly higher baseline values of FGF-b than those with liver only disease.

**Fig. 2:**
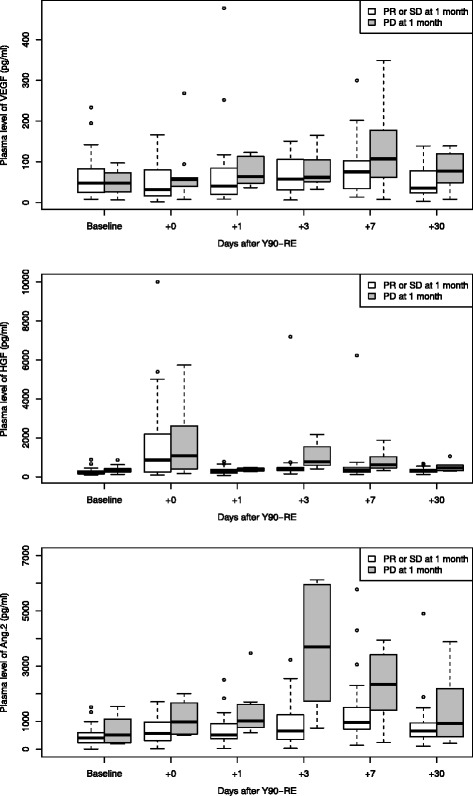
VEGF (*top*), HGF (*middle*) and angiopoietin-2 (*bottom*) levels per time point (baseline, +0, +1, +3, +7 and +30 days post treatment, respectively), for patients with and without response at 1 month post treatment, i.e. partial response and stable disease (*n* = 32) versus progressive disease (*n* = 10) of the liver

Following ^90^Y-RE, three of the investigated angiogenic factors demonstrated an overall increase in plasma levels, i.e. VEGF, HGF and Ang-2. Figure 2 shows the plasma values for these three angiogenic factors, stratified for patients with response (CR, PR or SD, *n* = 32) and those with progressive disease (*n* = 10) of the liver at 1-month follow-up. The linear mixed model analysis showed that time as a factor did not contribute to the model performance for FGF-b and SDF-1a, therefore excluding a significant change over time.

For VEGF, a slight overall rise in plasma level was observed at 7 days post treatment, though more pronounced in patients with progressive disease at 1 month after ^90^Y-RE. This time effect did contribute to the model performance; however, there was no statistically significant difference between the model with and without response as a fixed effect (*p* = 0.28). Liver dose did not contribute to the model performance either.

Plasma levels of HGF demonstrated a rise promptly after injection of the microspheres (blood drawn from the still indwelling sheath in the femoral artery). This holds true for both responders and non-responders. Yet, plasma levels of non-responders showed another peak, i.e. at 3 and 7 days after treatment, which was significantly different from responders (*p* = 0.007). Angiopoietin-2 levels were also statistically significantly higher at 3 and 7 days after treatment for patients with rapidly progressive disease, i.e. liver progression at 1-month follow-up, compared to those with disease control (*p* = 0.01). Model performances for HGF and angiopoietin-2 did not improve on adding received liver dose as a factor.

**Fig. 3:**
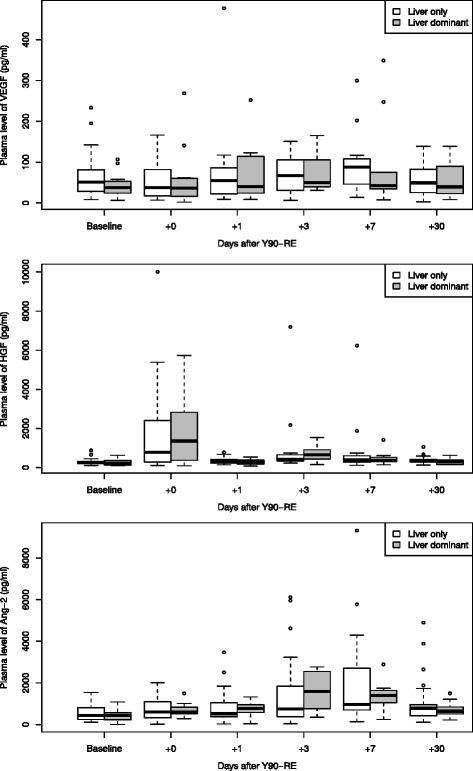
VEGF (*top*), HGF (*middle*) and angiopoietin-2 (*bottom*) levels per time point (baseline, +0, +1, +3, +7 and +30 days post treatment, respectively), for patients with liver only disease and (*n* = 30) versus patients with extrahepatic disease (*n* = 12)

**Fig. 4:**
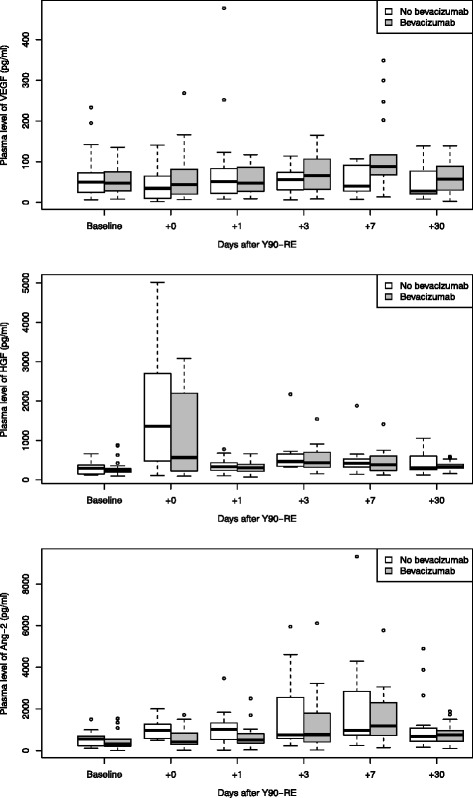
VEGF (*top*), HGF (*middle*) and angiopoietin-2 (*bottom*) levels per time point (baseline, +0, +1, +3, +7 and +30 days post treatment, respectively), for patients who had previously received bevacizumab (*n* = 25) and those who did not (*n* = 17)

In addition, time variation of angiogenic factors for two patient subgroups were analysed, i.e. (1) patients with extrahepatic disease versus patients without extrahepatic disease (patients with liver only disease) (Fig. 3) and (2) patients previously treated with bevacizumab versus those who had not received bevacizumab (Fig. 4). There were no statistically significant differences between the models with and without respectively EHD or use of bevacizumab as a fixed effect.

Furthermore, a statistically significant difference in median overall survival was demonstrated between early responders and non-responders, of 341 (224–458) days versus 160 (122–198) days respectively (*p* value 0.003) (Table 3). There was no difference in baseline value of HGF and Ang-2 between early responders and non-responders. No statistically significant differences were seen between the 10 patients with rapidly progressive disease at 1-month follow-up and the 32 patients with CR, PR or SD at 1-month follow-up with respect to a number of characteristics that can be assumed to be important influences on prognosis and response, i.e. liver tumour involvement, performance score, presence of extrahepatic disease and received liver dose (Table 4). The difference in Ang-2 levels at 3 and 7 days after treatment between responders and non-responders is still statistically significant when comparing patients based on response at three months post treatment.

**Table 4:** Responders (PR or SD) and non-responders (PD) at 1 month after treatment

	PR or SD (*n* = 32)	PD (*n* = 10)
WHO performance score (n)		
0	19	4
1	12	5
2	1	1
Extrahepatic disease at baseline (n)		
Yes	7	5
No	25	5
Tumour load (% of the liver)	12%	22%
Liver dose^a^ (Gy)	45	43

Several patient characteristics of the patients defined as responders (PR or SD) and those defined as non-responders (PD). No statistically significant differences were observed

*PR* partial response, *SD* stable disease, *PD* progressive disease, *Gy* gray

^a^Liver dose was calculated as (administrated activity(MBq))/(treated liver volume(ml)) x 50

## Discussion

Yttrium-90 radioembolization is an increasingly utilized treatment in clinical practice for unresectable and chemorefractory liver tumours. A large group of patients who can potentially benefit from this therapy are those with liver metastases from colorectal cancer [1–3, 5, 11]. Although the group of patients with CRCLM seems relatively homogeneous, response to treatment varies considerably. Probably, more heterogeneity between patients exists on a cellular level rather than on a macroscopic level, resulting in a difference in treatment response. At present, however, no definite evidence for predictive factors for treatment response of ^90^Y-RE has been identified, although for example presence of extrahepatic disease and performance status have been associated with survival [21, 22]. In clinical practice, we have observed that patients with EHD appear to have poorer response rates to ^90^Y-RE than those with liver only disease. Possibly, a systemic trigger of the angiogenic cascade underlies this phenomenon. With this hypothesis, we started a prospective study to investigate the angiogenic cascade following treatment with ^90^Y-RE.

The median overall survival in our group was 9.2 months. This is in accordance with recently published data on similar patient groups with heavily pre-treated advanced colorectal cancer, reporting median overall survival ranging from 8.3 to 11.9 months [21, 23–25]. Additionally, we focused first on the baseline levels of several classic angiogenic factors. Relatively large variations were found in baseline plasma levels of these angiogenic factors between patients. However, there was no apparent threshold that separated patients with early response from those with rapid progressive disease (at 1-month follow-up).

Secondly, we evaluated the changes in plasma levels of angiogenic factors after treatment in relation to treatment response at 1-month follow-up (Fig. 2). Statistically significant differences were found between patients with early disease control (CR, PR or SD) and those with rapidly progression (PD) at 1-month follow-up. Both HGF and angiopoietin-2 plasma levels showed a rise at three and seven days after treatment in non-responders, while hardly any changes were seen in responders. In principal, one would argue that the embolic effect of ^90^Y-RE, if any, would be the same in all patients, yet not all of our patients seem to have been equally susceptible to an upregulation of angiogenic factors. And at the same time, this upregulation of angiogenic factors is connected to early progressive disease and thus perhaps an early predictor of unfavourable response to treatment.

Furthermore, a statistically significant difference in median overall survival was observed between responders and non-responders at 1-month follow-up, 341 (224 – 458) versus 160 (122 – 198) days respectively. No statistically significant differences in baseline patient characteristics were observed between these responders and non-responders. Our findings suggest that ^90^Y-RE can cause an increase in circulating angiogenic factors, which may be predictive of rapid progressive disease.

Over the past decades, several studies have been conducted on systemic release of angiogenic factors after transarterial treatment of liver tumours. An overview of these papers is presented in Table 5. The first studies included patients with HCC treated with either chemoembolization (TACE) or bland embolization (TAE) and the focus of angiogenic analysis was on VEGF [8–10, 26]. Suzuki et al. measured serum levels of HGF and VEGF at 1, 3 and 7 days after bland embolization of HCC. They observed a rise in VEGF at day 7 but did not demonstrate a subsequent fall in VEGF nor did they evaluate tumour response [9]. Korse et al. included 12 patients with neuroendocrine tumours into their study and followed VEGF and endothelin levels during the first 8 days after bland embolization [27]. Unfortunately, they too did not include tumour response into their study. Sergio et al. analysed VEGF and b-FGF and the association with response at one month after TACE. Response was not scored according to (modified) RECIST, but expressed as the percentage of residual activity at CT imaging, and responders were defined as those with an ablation rate between 70–100 % and non-responders as those with residual activity higher than 30 %. Even though all 71 patients demonstrated an increase in VEGF level at 30-day follow-up, non-responders showed the highest increase [10].

**Table 5:** Overview of previous studies on angiogenic factors and transarterial treatment of liver tumours

Author	Year	Patients	Treatment	Factors	Samples collected	Results
Carpizo et al. [28]	2014	*n* = 15 CRCLM	^90^Y-RE	VEGF, Ang-2, b-FGF, PDGF-BB, TSP-1, follistatin, leptin, IL-8	Baseline	* Transient increases in many angiogenic cytokines * Some changes associated with worse OS
		*n* = 7 HCC			6 h, and 3, 14, 30, 60, 90 and 120 days of follow-up	
Korse et al. [27]	2011	*n* = 12 NET	HAE	VEGF, ET-1, proET-1	Baseline	* VEGF and proET-1 showed temporarily increase after treatment
					1, 2, 3, 4, 5, 6, 7 and 8 days of follow-up	
Sergio et al. [10]	2008	*n* = 71 HCC	TACE	VEGF, b-FGF, uPA	Baseline	* VEGF levels were higher in non-responders at 1-month follow-up
					3 and 30 days of follow-up	* Below-median VEGF levels predicted a longer survival
Shim et al. [26]	2008	*n* = 147 HCC	TACE	VEGF	Baseline	* High increment in serum VEGF level 1–2 days post treatment was associated with distant metastasis and unfavourable outcomes
					1–2 and 30 days of follow-up	
Li et al. [8]	2004	*n* = 45 HCC	TACE	VEGF	Baseline	* A high pre-treatment VEGF level was associated with poor response
		*n* = 20 benign disease			1, 3, 7 and 30 days of follow-up	
		*n* = 17 healthy controls				* VEGF levels increased significantly on the first day post treatment
Suzuki et al. [9]	1999	*n* = 38 HCC	TAE	VEGF, HGF	Baseline	* No significant alterations in HGF levels
					1, 3 and 7 days of follow-up	* VEGF levels increased significantly at 7 days post treatment

*CRCLM* colorectal cancer liver metastases, *HCC* hepatocellular carcinoma, ^*90*^ *Y-RE* yttrium-90 radioembolization, *VEGF* vascular endothelial growth factor, *Ang-2* angiopoietin-2, *b-FGF* basic fibroblast growth factor, *PDGF-BB* platelet-derived growth factor subunit BB, *TSP-1* thrombospondin-1, *IL-8* interleukin-8, *NET* neuroendocrine tumours, *HAE* hepatic artery embolization, *ET-1* endothelin-1, *proET-1* proendothelin-1, *TACE* transarterial chemoembolization, *uPA* urokinase-type plasminogen activator, *TAE* transarterial embolization, *HGF* hepatocyte growth factor

Recently, Carpizo et al. [28] have conducted a pilot study on angiogenic factors and ^90^Y-RE. They included 15 patients with CRCLM and 7 patients with HCC and measured several angiogenic factors, including non-classical factors such as interleukin-8, at baseline and during follow-up until 120 days after treatment. As in our present study, they too demonstrated rises in VEGF and Ang-2 after treatment, even though their sample size was rather limited. Unfortunately, they did not report tumour response in relation to the levels of angiogenic factors, but only survival data. Median overall survival for the entire patient group was 8 months, but no separate values for CRCLM and HCC patients were reported.

Meanwhile, the early progression of liver lesions in 10 patients largely consists of the appearance of new liver lesions (*n* = 8/10), and not of the growth of (non)target lesions. Target lesions, in fact, were stable in all 10/10 patients. This supports our hypothesis that an increase in circulating angiogenic factors may induce growth of previously invisible hepatic micro-metastases. Even though these new appearing lesions are located in the treated organ, the largest arterial flow at the time of treatment will have been to the macroscopic metastases, leaving the micro metastases (and of course the normal liver parenchyma) relatively untreated and susceptible to growth stimulating factors. Perhaps, the concomitant use of an anti-angiogenic agent could counteract this phenomenon. Gorski et al. have described the relationship between VEGF and the anti-tumour effects of ionizing radiation [29]. They report that VEGF expression is induced in Lewis lung carcinomas both in vitro and in vivo after exposure to ionizing radiation. Furthermore, they demonstrated that treatment of tumour-bearing mice with a neutralizing antibody to VEGF prior to irradiation was associated with a greater than additive anti-tumour effect. With this, they emphasize the potential importance of combining radiation therapy with systemic anti-angiogenic treatment to increase anti-tumour effects.

In addition to the above described increase in HGF at days 3 and 7 after treatment in non-responders, a large rise in HGF directly after microsphere injection (approximately 5 min after end of injection) was observed for all patients. This may be related to the angiographic procedure itself, with catheter manipulation in the hepatic arteries, giving rise to this release of hepatocyte growth factor. One day after treatment, HGF levels have returned approximately to baseline. During follow-up, patients with PR or SD at 1 month after treatment did not experience any other rises in HGF plasma levels during the first 30 days, contrary to patients with early PD. Suzuki et al. also investigated HGF levels but did not find this large increase, because their first follow-up sample was collected 1 day after treatment instead of directly after microsphere injection [9].

Survival analysis showed a remarkable difference in time to liver progression between patients with and without extrahepatic disease at baseline. Obviously, ^90^Y-RE is a liver-directed treatment and any extrahepatic lesions will not be treated when no systemic therapy is added. However, in our cohort, patients with extrahepatic lesions had earlier progression of liver lesions than those with liver only disease at baseline. Perhaps this reflects a more aggressive tumour type that is less susceptible to radiation damage as well as more prone to disseminate throughout the body. This may be an additional reason to primarily select patients with liver only disease for ^90^Y-RE as stand-alone treatment, as they may benefit more with respect to their liver lesions, on top of the fact that extrahepatic lesions would be left untreated. For patients with extrahepatic lesions, a combination of ^90^Y-RE and systemic treatment is perhaps more suitable [2, 30]. One of the next steps for this would be a safety study on the concomitant use of bevacizumab with ^90^Y-RE.

An important limitation of our study was the relatively small sample size that did not allow development of a clinical prediction model given the limited number of predictors that can be included into a model with this sample size. In the following study a larger patient population should be included. Advantages of our study were its prospective design that ensured standardization of study procedures and the homogeneity of our study population, consisting of only patients with unresectable colorectal cancer liver metastases who had previously received at least 1 regimen of systemic treatment. Furthermore, we have created a special protocol for blood collection and processing to ensure that platelet free plasma was produced and all plasma samples were stored at −80 °C within 60 min, to avoid breakdown of the angiogenic proteins, to which mainly VEGF is prone at room temperature. In addition, our study was the first and largest cohort study to investigate circulating angiogenic factors after ^90^Y-RE for CRCLM in relation to treatment response according to RECIST.

## Conclusions

We showed for the first time that a significant rise in plasma levels of HGF and Ang-2 at 3 and 7 days post ^90^Y-RE treatment was associated with progressive liver disease at 1-month follow-up. Moreover, these early non-responders have significantly worse overall survival compared to responders, i.e. 5.2 versus 11.1 months. In addition, our data showed that the presence of extrahepatic disease at baseline is a predictor for early disease progression of the tumours in the liver.

Our results may open the discussion for more stringent patient selection for ^90^Y-RE, with respect to for example extrahepatic disease, and combining ^90^Y-RE with systemic anti-angiogenetic treatment for better treatment response in CRCLM salvage patients.

## References

[1] Gray B, Van Hazel GA, Hope M (2001). Randomised trial of SIR-Spheres® plus chemotherapy vs. chemotherapy alone for treating patients with liver metastases from primary large bowel cancer. Ann Oncol.

[2] Hendlisz A, Eynde MV, Peeters M (2010). Phase III trial comparing protracted intravenous fluorouracil infusion alone or with yttrium-90 resin microspheres radioembolization for liver-limited metastatic colorectal cancer refractory to standard chemotherapy. J Clin Oncol.

[3] Van Hazel G, Blackwell A, Anderson J (2004). Randomised phase 2 trial of SIR-Spheres® plus fluorouracil/leucovorin chemotherapy versus fluorouracil/leucovorin chemotherapy alone in advanced colorectal cancer. J Surg Oncol.

[4] Kennedy AS, Coldwell D, Nutting C (2006). Resin 90Y-microsphere brachytherapy for unresectable colorectal liver metastases: modern USA experience. Int J Radiat Oncol Biol Phys.

[5] Vente MA, Wondergem M, van der Tweel I (2009). Yttrium-90 microsphere radioembolization for the treatment of liver malignancies: a structured meta-analysis. Eur Radiol.

[6] Scheer MG, Stollman TH, Vogel WV, Boerman OC, Oyen WJ, Ruers TJ (2008). Increased metabolic activity of indolent liver metastases after resection of a primary colorectal tumor. J Nucl Med.

[7] Venderbosch S, de Wilt JH, Teerenstra S (2011). Prognostic value of resection of primary tumor in patients with stage IV colorectal cancer: retrospective analysis of two randomized studies and a review of the literature. Ann Surg Oncol.

[8] Li XFGS, Zheng CS, Zhuo CK, Liu X (2004). Expression of plasma vascular endothelial growth factor in patients with hepatocellular carcinoma and effect of transcatheter arterial chemoembolization therapy on plasma vascular endothelial growth factor level. World J Gastroenterol.

[9] Suzuki HMM, Kawaguchi C, Adachi M, Miura S, Ishii H (1999). Serum vascular endothelial growth factor in the course of transcatheter arterial embolization of hepatocellular carcinoma. Int J Oncol.

[10] Sergio A, Cristofori C, Cardin R (2008). Transcatheter arterial chemoembolization (TACE) in hepatocellular carcinoma (HCC): the role of angiogenesis and invasiveness. Am J Gastroenterol.

[11] Rosenbaum CE, Verkooijen HM, Lam MG (2013). Radioembolization for treatment of salvage patients with colorectal cancer liver metastases: a systematic review. J Nucl Med.

[12] Dvorak HF (2002). Vascular permeability factor/vascular endothelial growth factor: a critical cytokine in tumor angiogenesis and a potential target for diagnosis and therapy. J Clin Oncol.

[13] Rahbari NN, Reissfelder C, Muhlbayer M (2011). Correlation of circulating angiogenic factors with circulating tumor cells and disease recurrence in patients undergoing curative resection for colorectal liver metastases. Ann Surg Oncol.

[14] Carmeliet P, Jain RK (2000). Angiogenesis in cancer and other diseases. Nature.

[15] Giammarile F, Bodei L, Chiesa C (2011). EANM procedure guideline for the treatment of liver cancer and liver metastases with intra-arterial radioactive compounds. Eur J Nucl Med Mol Imaging.

[16] Salem R, Thurston KG (2006). Radioembolization with yttrium-90 microspheres: a state-of-the-art brachytherapy treatment for primary and secondary liver malignancies: part 3: comprehensive literature review and future direction. J Vasc Interv Radiol.

[17] Salem R, Thurston KG (2006). Radioembolization with 90yttrium microspheres: a state-of-the-art brachytherapy treatment for primary and secondary liver malignancies. Part 2: special topics. J Vasc Interv Radiol.

[18] Salem R, Thurston KG (2006). Radioembolization with 90Yttrium microspheres: a state-of-the-art brachytherapy treatment for primary and secondary liver malignancies. Part 1: Technical and methodologic considerations. J Vasc Interv Radiol.

[19] de Jager W, Hoppenreijs EP, Wulffraat NM, Wedderburn LR, Kuis W, Prakken BJ (2007). Blood and synovial fluid cytokine signatures in patients with juvenile idiopathic arthritis: a cross-sectional study. Ann Rheum Dis.

[20] De Jager W, Prakken BJ, Bijlsma JW, Kuis W, Rijkers GT (2005). Improved multiplex immunoassay performance in human plasma and synovial fluid following removal of interfering heterophilic antibodies. J Immunol Methods.

[21] Bester L, Meteling B, Pocock N (2012). Radioembolization versus standard care of hepatic metastases: comparative retrospective cohort study of survival outcomes and adverse events in salvage patients. J Vasc Interv Radiol.

[22] Lim L, Gibbs P, Yip D (2005). Prospective study of treatment with selective internal radiation therapy spheres in patients with unresectable primary or secondary hepatic malignancies. Intern Med J.

[23] Seidensticker R, Denecke T, Kraus P (2012). Matched-pair comparison of radioembolization plus best supportive care versus best supportive care alone for chemotherapy refractory liver-dominant colorectal metastases. Cardiovasc Intervent Radiol.

[24] Martin LK, Cucci A, Wei L (2012). Yttrium-90 radioembolization as salvage therapy for colorectal cancer with liver metastases. Clin Colorectal Cancer.

[25] Fahmueller YN, Nagel D, Hoffmann RT (2012). Predictive and prognostic value of circulating nucleosomes and serum biomarkers in patients with metastasized colorectal cancer undergoing Selective Internal Radiation Therapy. BMC Cancer.

[26] Shim JH, Park JW, Kim JH (2008). Association between increment of serum VEGF level and prognosis after transcatheter arterial chemoembolization in hepatocellular carcinoma patients. Cancer Sci.

[27] Korse CM, Bonfrer JM, Prevoo W, Baas P, Taal BG (2011). Increase of angiogenic growth factors after hepatic artery embolization in patients with neuroendocrine tumours. Tumour Biol.

[28] Carpizo DR, Gensure RH, Yu X (2014). Pilot study of angiogenic response to yttrium-90 radioembolization with resin microspheres. J Vasc Interv Radiol.

[29] Gorski DHBM, Jaskowiak NT, Calvin DP, Mauceri HJ, Salloum RM, Seetharam S, Koons A, Hari DM, Kufe DW, Weichselbaum RR (1999). Blockade of the vascular endothelial growth factor stress response increases the antitumor effects of ionizing radiation. Cancer Res.

[30] Gibbs P, Heinemann V, Findlay MPN (2015). SIRFLOX: Randomized phase III trial bevacizumab (bev) versus mFOLFOX6 + selective internal radiation therapy (SIRT) in patients with metastatic colorectal cancer (mCRC). J Clin Oncol.

